# The effect of triglycerides to high-density lipoprotein cholesterol ratio on the reduction of renal function: findings from China health and retirement longitudinal study (CHARLS)

**DOI:** 10.1186/s12944-021-01542-5

**Published:** 2021-09-20

**Authors:** Shiqi Lv, Han Zhang, Jing Chen, Ziyan Shen, Cheng Zhu, Yulu Gu, Xixi Yu, Di Zhang, Yulin Wang, Xiaoqiang Ding, Xiaoyan Zhang

**Affiliations:** 1 Department of Nephrology, Zhongshan Hospital, Fudan University, No. 180 Fenglin Road, 200032 Shanghai, China; 2 Shanghai Medical Center of Kidney Disease, 200032 Shanghai, China; 3 Shanghai Institute of Kidney and Dialysis, No. 136 Medical College Road, 200032 Shanghai, China; 4 Shanghai Key Laboratory of Kidney and Blood Purification, 200032 Shanghai, China

**Keywords:** Estimated glomerular filtration rate (eGFR), Triglycerides to high-density lipoprotein cholesterol ratio (TG/HDL-C), China Health and Retirement Longitudinal Study (CHARLS)

## Abstract

**Background:**

Previous studies show that abnormal lipoprotein metabolism can increase the prevalence of chronic kidney disease (CKD). This study prospectively investigated the association of triglycerides to high-density lipoprotein cholesterol (TG/HDL-C) ratio and renal dysfunction in the Chinese population.

**Methods:**

This longitudinal cohort research examined 7,316 participants (age range: 22–93) from the China Health and Retirement Longitudinal Study (CHARLS), including 6,560 individuals with estimated glomerular filtration rate (eGFR) ≥ 60 mL/min/1.73 m^2^ (normal renal function, NRF) group and 756 with eGFR < 60 mL/min/1.73 m^2^ (impaired renal function, IRF) group. In NRF group, reduction in renal function was defined as eGFR < 60 mL/min/1.73 m^2^ at exit visit and in IRF group, it was defined as decline in eGFR category, average eGFR decline > 5 mL/min/1.73 m^2^ per year or > 30 % decrease in eGFR from baseline.

**Results:**

The study results showed that TG/HDL-C ratio was positively associated with the risk of renal function decline in the NRF group (OR 1.30, 95 %CI 1.03–1.65, *P* = 0.03) and the IRF group (OR 1.90, 95 %CI 1.21–3.23, *P* = 0.02) when adjusting for age, gender, obesity, diabetes, hypertension, waist circumference, drinking, smoking, history of heart disease and stroke, low-density lipoprotein cholesterol and eGFR category. Analysis of the IRF group indicated that relative to the group of TG/HDL-C < 1.60, the group of TG/HDL-C ≥ 2.97 had an increased risk for the decline of eGFR category (OR 1.89, 95 %CI 1.12–3.21, *P* = 0.02) and > 30 % decline in eGFR (OR 2.56, 95 %CI 1.05–6.38, *P* = 0.04).

**Conclusions:**

The high TG/HDL-C ratio was an independent risk factor for declining renal function in the Chinese population.

**Supplementary Information:**

The online version contains supplementary material available at 10.1186/s12944-021-01542-5.

## Introduction

The prevalence of chronic kidney disease (CKD) gradually increase during the past decade and is gradually aroused global public attention [1]. The estimated glomerular filtration rate (eGFR), which is recognized as the effective indicator of renal excretory function, is the most important diagnostic and prognostic indicator of CKD. The morbidity and mortality of end-stage renal disease (ESRD) and cardiovascular disease (CVD) increase with the eGFR decline [2]. Early identification and appropriate management of the risk factors associated with eGFR decline may reduce CKD incidence and prevalence and prevent ESRD and CVD.

Abnormal lipoprotein metabolism, as indicated by a high level of triglyceride (TG) or a low level of high-density lipoprotein cholesterol (HDL-C), is a possible risk factor for CKD [3–5]. These lipid abnormalities are significant in increasing the morbidity from CKD, particularly in patients who have type 2 diabetes [6–8]. Several studies reported triglycerides to high-density lipoprotein cholesterol (TG/HDL-C) ratio was positively associated with insulin resistance, and compared with any other lipid parameters, the prediction power to cardiovascular events was better [9–12]. Several cross-sectional studies reported higher TG/HDL-C levels increasing the prevalence of CKD [13–16].

A national survey of China estimated 119.5 million patients with CKD, more than any other country [17]. Other research estimated that the overall prevalence of dyslipidemia in China was 41.9 % [18]. These findings indicated the need for further research on the relationship between dyslipidemia and CKD. The effect of dyslipidemia on eGFR decline is unknown, and in the Chinese population, the published data on the association between these two abnormalities are limited. This study investigated the effect of the TG/HDL-C on eGFR decline in adults from the China Health and Retirement Longitudinal Study (CHARLS), a 4-year prospective longitudinal study.

## Methods

### Study population

**Fig. 1:**
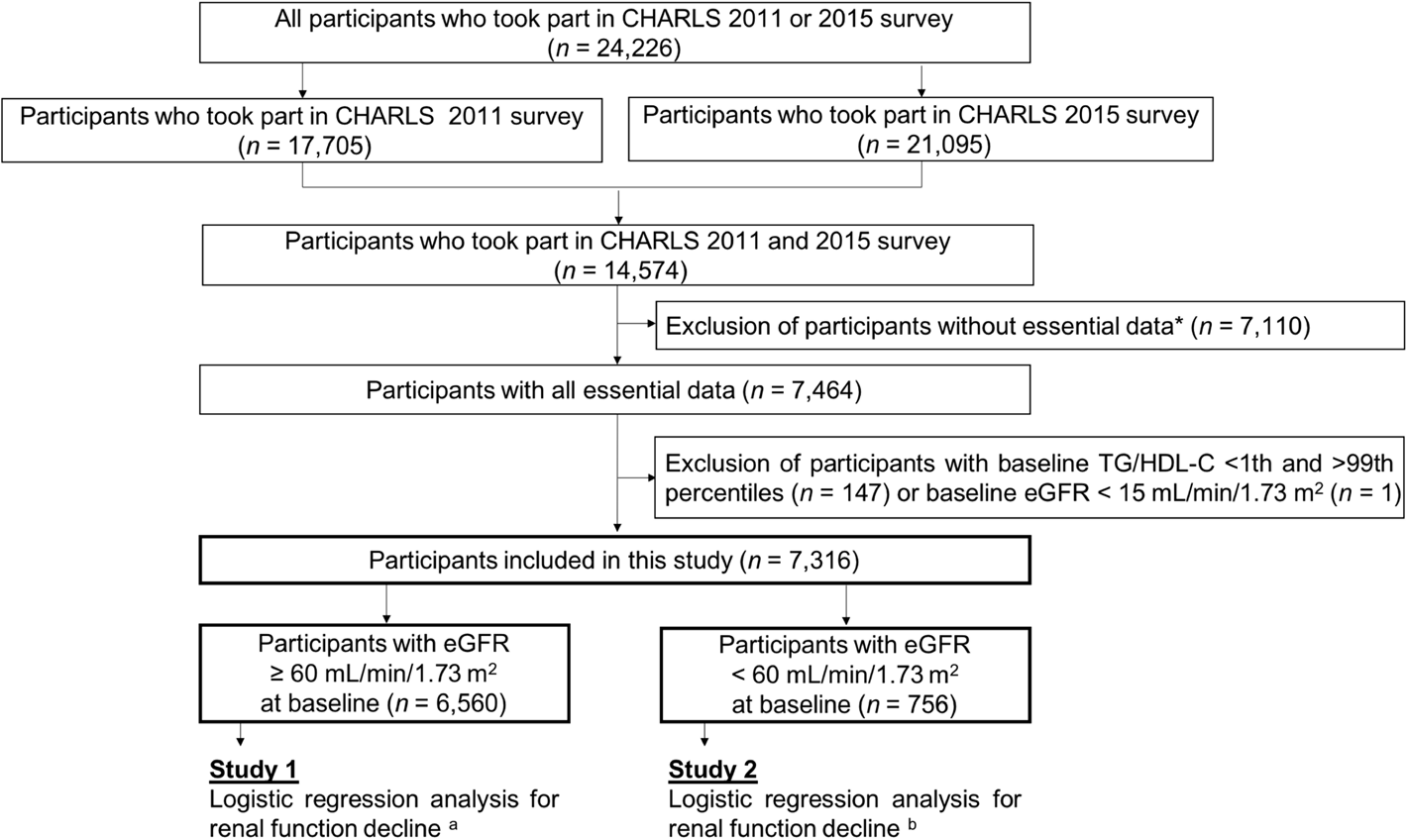
Flowchart of CHARLS and the enrollment of the study. CHARLS, China Health and Retirement Longitudinal Study. *Essential data: age, gender, TG, HDL-C and eGFR levels. ^a^ Renal function decline was defined as eGFR < 60 mL/min/1.73 m^2^ at exit visit. ^b^ Renal function decline was defined as decline in eGFR category, average eGFR decline > 5 mL/min/1.73 m^2^ per year or > 30 % decrease in eGFR from baseline

All data were from the CHARLS, a longitudinal survey mainly focusing on the middle-aged and elderly population. Baseline data were from 2011 to 2012, and the outcome was recorded in 2015. A total of 150 county-level units were randomly selected from the 28 provinces and autonomous regions in mainland China using the probability-proportional-to-size sampling technique. Primary sampling units (villages or communities) within each county-level unit were randomly chosen using the same method. Previous studies reported the CHARLS objectives, design, and methods detailly [19, 20]. Finally, 24,266 individuals from 10,257 households in 150 counties were stratified and randomly selected to receive the survey in either 2011 or 2015. The 14,574 individuals who participated in both two surveys were enrolled. The 7,110 individuals with unavailable essential data include age, gender, TG, HDL-C, and eGFR levels, 147 individuals with baseline TG/HDL-C < 1 and > 99th percentiles as statistical outliers, and one individual with eGFR < 15 mL/min/1.73 m^2^ in 2011 were excluded. Finally, a total of 7,316 participants (3,315 men and 4,001 women) who were 22 to 93 years old with eGFR from 20 to 122 mL/min/1.73 m^2^ were recruited in the research (Fig. 1).

### Sample collection

Trained staff collected the venous blood samples in overnight fasting participants. Then the samples were transported to the local laboratory timely and stored at 4 °C. The blood samples were centrifugated and stored at − 20 °C before been transported to the central laboratory in Beijing and frozen at − 80 °C before analysis. All study laboratories had the standardized certification.

### Definition of eGFR and TG/HDL-C

The Jaffe creatinine method was used to measure serum creatinine (SCr). This study used coefficient-modified CKD Epidemiology Collaboration (CKD-EPI) equation from Japanese [21] to calculate eGFR (mL/min/1.73m^2^) = 0.813 × 141 × *min* (SCr/κ, 1) ^α^ × *max* (SCr/κ, 1)^−1.209^ × 0.993 ^Age^ × 1.021 [if female] × 1.159 [if black], where κ is 0.7 for females and 0.9 for males, α is − 0.329 for females and − 0.411 for males, *min ()* indicates the minimum between SCr/κ and 1, and *max ()* indicates the maximum between SCr/κ and 1. According to kidney disease guideline in 2012, individuals with eGFR < 60mL/min/1.73 m^2^ can be diagnosed as CKD [22]. eGFR category was defined as G1 ≥ 90, 60 ≤ G2 < 90, 45 ≤ G3a < 60, 30 ≤ G3b < 45, 15 ≤ G4 < 30, G5 < 15mL/min/1.73 m^2^.

The enzymatic colorimetric test method was used to measure TG, HDL-C, and low-density lipoprotein cholesterol (LDL-C). TG (mg/dL) divided by HDL-C (mg/dL) was used in this study to calculate the TG/HDL-C ratio. Based on the TG/HDL-C ratio, participants were classified into tertiles: group 1, TG/HDL-C < 1.60; group 2, 1.60 ≤ TG/HDL-C < 2.97; group 3, TG/HDL-C ≥ 2.97.

### Assessment of covariates

All participants were collected medical history (hypertension, hyperglycemia or diabetes, stroke, and heart diseases) and lifestyle information (drinking and smoking) during face-to-face interviews by trained interviewers. The definition of hypertension was blood pressure over 140/90 mmHg (mean of 3 measurements) or a history of hypertension. The definition of diabetes was glycated fasting blood glucose ≥ 7.0 mmol/L, hemoglobin A1c (HbA1c) ≥ 6.5 %, or self-reported history of diabetes or high blood sugar. The definition of the history of heart diseases was the individuals had been diagnosed with myocardial infarction, coronary heart disease, congestive heart failure, or other heart diseases. The definition of drinking was drinking alcoholic more than once a month. The definition of smoking was smoking more than 100 cigarettes in the past. The trained staff collected the height, weight, and waist circumference data of all participants. The body mass index (BMI) was calculated as weight/height^2^ (kg/m^2^). Obesity used BMI to classify participants as underweight (BMI < 18.5), normal weight (18.5 ≤ BMI < 24), overweight (24 ≤ BMI < 28), or obesity (BMI ≥ 28 kg/m^2^).

### Study protocol

Individuals in this study were divided into two groups by baseline eGFR (normal renal function [NRF]: eGFR ≥ 60 or impaired renal function [IRF]: eGFR < 60 mL/min/1.73 m^2^), and examined the relationship of TG/HDL-C ratio and renal dysfunction in these two subgroups, respectively (Table 1).

**Table 1:** Eligibility of participants, sample size, and definition of outcome in each study

	Eligibility of participants	Sample Size	Definition of Outcome
Subgroup 1	eGFR ≥ 60 mL/min/1.73 m^2^ at baseline	6,560	eGFR less than 60 mL/min/1.73 m^2^ at the exit visit in 2015
Subgroup 2	eGFR < 60 mL/min/1.73 m^2^ at baseline	756	Composite endpoint of decline in eGFR category^b^, Rapid decline in eGFR^c^ and > 30 % decline in eGFR^d^

Abbreviations: *eGFR* estimated glomerular filtration rate; *CKD* chronic kidney disease.

^a^Decline in eGFR category (≥ 90 [G1], 60-89 [G2], 45-59 [G3a], 30-44 [G3b], 15-29 [G4], < 15 [G5] mL/min/1.73 m2) was defined as certain decrease in eGFR category from baseline.

^b^Rapid decline in eGFR was defined as sustained decline in eGFR > 5 mL/min/1.73 m2 per year.

^c^30% decline in eGFR was defined as >30% decrease in eGFR from baseline.

#### Study 1

For the 6,560 participants with eGFR ≥ 60 mL/min/1.73 m^2^ in NRF group at baseline, renal function decline was defined as eGFR < 60 mL/min/1.73 m^2^ in 2015 [23].

#### Study 2

For the 756 participants with 15 ≤ eGFR < 60 mL/min/1.73 m^2^ in IRF group at baseline, the definition of renal function decline was decline in eGFR category from baseline, > 5 mL/min/1.73 m^2^ per year or > 30 % decrease in eGFR from baseline. The definition of decline in the eGFR category was a certain decline in the eGFR category compared with baseline. The definition of rapid decline in eGFR was an annual decrease in eGFR > 5 mL/min/1.73 m^2^. The definition of > 30 % decline in eGFR was > 30 % decrease in eGFR compared with baseline.

### Statistical Analysis

This study used Stata/MP version 14.1 (StataCorp, College Station, TX, USA) for statistical analysis. Kruskal-Wallis test and Chi-square test were used for analysis of continuous and categorical variables, respectively. The logistic regression model was used to explore the association of the TG/HDL-C and the decline in renal function. The confounding covariates adjusted in the multivariate-adjusted model including age, gender, waist circumference, diabetes, hypertension, obesity, drinking, smoking, stroke or heart diseases history, LDL-C, and eGFR category.

## Results

### Baseline characteristics of participants

This study enrolled 7,316 participants (3,315 men and 4,001 women) aged 22 to 93. Baseline features are presented in Table 2. Using the trisection of TG/HDL-C as cut-point, the participants were divided into three group: group 1 (TG/HDL-C < 1.60, n = 2,453), group 2 (1.60 ≤ TG/HDL-C < 2.97, n = 2,423), and group 3 (TG/HDL-C ≥ 2.97, *n* = 2,440). The participants with eGFR < 60 mL/min/1.73 m^2^ in group 1, group 2, and group 3 were 9.13 %, 9.12 %, and 12.75 % respectively. Similarly, BMI, waist circumference, the prevalence of hypertension, diabetes, obesity, and heart diseases increased with the higher TG/HDL-C levels. Moreover, the association between TG/HDL-C and the levels of HbA1c, TG, and total cholesterol (TC) was positively and was negative with the level of HDL-C.

**Table 2:** Baseline characteristics of study population by TG/HDL-C ratio

Variable	Group 1	Group 2	Group 3	*P*
	**TG/HDL-C < 1.60**	**1.60 ≤ TG/HDL-C < 2.97**	**TG/HDL-C ≥ 2.97**	
Number, *n (%)*	2,453 (33.53)	2,423 (33.12)	2,440 (33.35)	
Age, mean *(SD)*, years	59.31 (9.40)	58.68 (8.93)	58.16 (8.75)	< 0.001
Women, *n (%)*	1,265 (51.57)	1,347 (55.59)	1,389 (56.93)	< 0.001
BMI, mean *(SD)*, kg/m^2^	22.31 (3.56)	23.59 (3.70)	25.10 (3.82)	< 0.001
Obesity, *n (%)*	383 (15.61)	543 (22.41)	757 (31.02)	< 0.001
Waist circumference, mean *(SD)*, cm	80.80 (10.62)	84.49 (12.62)	88.48 (12.83)	< 0.001
Hypertension, *n (%)*	762 (31.13)	919 (37.96)	1,169 (48.07)	< 0.001
Diabetes, *n (%)*	242 (9.87)	327 (13.50)	607 (24.88)	< 0.001
Smoking, *n (%)*	999 (40.83)	888 (36.69)	901 (37.09)	0.005
Drinking, *n (%)*	857 (35.01)	687 (28.39)	669 (27.55)	< 0.001
History of stroke, *n (%)*	40 (1.64)	58 (2.40)	55 (2.27)	0.14
History of heart diseases, *n (%)*	239 (9.82)	280 (11.65)	380 (15.73)	< 0.001
LDL-C, mean *(SD)*, mg/dL	113.62 (29.99)	121.35 (34.19)	115.33 (36.93)	< 0.001
HDL-C, mean *(SD)*, mg/dL	63.27 (13.20)	50.05 (9.45)	38.89 (8.21)	< 0.001
TG, mean *(SD)*, mg/dL	68.88 (16.70)	108.85 (23.69)	209.90 (87.17)	< 0.001
TC, mean *(SD)*, mg/dL	188.28 (34.15)	191.16 (37.71)	198.23 (39.61)	< 0.001
TG/HDL-C, mean *(SD)*	1.12 (0.28)	2.20 (0.39)	5.72 (3.08)	< 0.001
Hemoglobin A1c, mean *(SD), (%)*	5.16(0.62)	5.22(0.73)	5.39(0.96)	< 0.001
Serum creatinine, mean *(SD)*, mg/dL	0.76 (0.17)	0.77 (0.18)	0.79 (0.19)	< 0.001
eGFR, mean *(SD)*, ml/min per 1.73m^2^	76.22 (11.17)	75.86 (11.35)	74.44 (12.23)	< 0.001
eGFR < 60 mL/min/1.73 m^2^, *n (%)*	224 (9.13)	221 (9.12)	311 (12.75)	< 0.001

Continuous variables were expressed as mean ± standard deviation (SD), and categorical variables were described as frequencies and percentages. Continuous variables were compared by Kruskal-Wallis test. Categorical variables were compared by Chi-square test. *BMI* body mass index; *SBP* systolic blood pressure; *DBP* diastolic blood pressure; *eGFR* estimated glomerular filtration rate; *HDL-C* high-density lipoprotein cholesterol; *LDL-C* low-density lipoprotein cholesterol; *TG* triglycerides; *TC* total cholesterol

A comparison of the NRF and IRF group are presented in Table S1. In these two groups, age, waist circumference, obesity, hypertension, diabetes, smoking, history of stroke and heart diseases, HbA1c, TG, LDL-C, TC, TG/HDL-C, SCr, and eGFR were significantly different (*P* < 0.05).

### The effect of TG/HDL-C ratio on incident CKD in participants with eGFR ≥ 60 mL/min/1.73 m^2^ at baseline

This part of the study examined the relationship between TG/HDL-C tertile with renal function decline in the NRF group. The clinical features of 6,560 NRF participants in the different TG/HDL-C tertiles were the same as those of all participants in Table 1 (Table S2). The OR for renal function decline in the NRF groups increased as the TG/HDL-C increased. Renal function decline increased in group 3 (OR 1.29, 95 % CI 1.05–1.59, *P* = 0.02) after adjusted age and gender using group 1 as the reference. The multivariate-adjusted model, which further adjusted for hypertension, diabetes, obesity, waist circumference, smoking, drinking, history of heart disease and stroke, LDL-C and eGFR category, indicated the risk for renal function decline was increased in group 2 (OR 1.27, 95 %CI 1.01–1.59, *P* = 0.04) and in the group3 (OR 1.30, 95 %CI 1.03–1.65, *P* = 0.03) (Table 3).

**Table 3:** The effect of TG/HDL-C ratio on renal function decline in participants with eGFR ≥ 60 mL/min/1.73 m^2^ at baseline

Study 1	Group 1	Group 2	Group 3
	**TG/HDL-C < 1.60** **(** ***N*** ** = 2,229)**	**1.60 ≤ TG/HDL-C < 2.97** **(** ***N*** ** = 2,202)**	**TG/HDL-C ≥ 2.97** **(** ***N*** ** = 2,129)**
Renal function decline, *n (%)*	208 (9.33)	223 (10.13)	217 (10.19)
Unadjusted, OR (95 % CI)	ref.	1.09 (0.90, 1.34)	1.10 (0.90, 1.35)
*P*		0.37	0.34
Age and gender adjusted, OR (95 % CI)	ref.	1.19 (0.97, 1.46)	1.29 (1.05, 1.59)
*P*		0.09	0.02
^a^ Multivariable-adjusted, OR (95 % CI)	ref.	1.27 (1.01, 1.59)	1.30 (1.03, 1.65)
*P*		0.04	0.03

*P* for trend for age and gender adjusted and multivariable-adjusted models are *P* < 0.05

^a^ Multivariable analysis was adjusted for age, gender, obesity, waist circumference, hypertension, diabetes or high blood sugar, smoking, drinking, history of heart disease and stroke, low-density lipoprotein cholesterol, eGFR category

### The effect of TG/HDL-C ratio on rapid eGFR decline in participants with eGFR < 60 mL/min/1.73 m^2^ at baseline

The TG/HDL-C with renal function decline in the IRF group was further examined in this part of the study. The clinical features of these 756 participants in the different TG/HDL-C tertiles were the same as those of all participants (Table 2) except for gender, hypertension, smoking, drinking, history of heart diseases, SCr, and eGFR, which were not statistically significant among those three tertiles (Table S3). The current study also examined the association between different components of renal function decline and the TG / HDL-C. The unadjusted logistic regression analysis indicated no association between renal function decline and TG/HDL-C (Table 4). However, after adjusted age and gender, relative to group 1, there was an increased risk in group 3 to the composite endpoint of renal function decline (OR 1.69, 95 %CI 1.07–2.68, *P =* 0.02). The results were similar in the multivariable-adjusted models (OR 1.90, 95 %CI 1.21–3.23, *P* = 0.02). There was also an association between different components of renal function decline and the TG/HDL-C. In particular, relative to the group 1, the group 3 had a significantly increased risk for decline in eGFR category (OR 1.89, 95 % CI 1.12–3.21, *P* = 0.02) and > 30 % decline in eGFR (OR 2.56, 95 % CI 1.05–6.38, *P* = 0.04) (Table 4). However, the rapid decline in eGFR did not achieve statistical significance (OR 3.28, 95 % CI 0.97–11.10, *P* = 0.06) (Table 4). These data suggested that the effect of TG/HDL-C ratio on renal function decline is consistent with its effect on most renal function decline components.

**Table 4:** The effect of TG/HDL-C ratio on renal function decline in participants with eGFR < 60 mL/min/1.73 m^2^ at baseline

	Group 1	Group 2	Group 3
	**TG/HDL-C < 1.60** **(*N* = 224)**	**1.60 ≤ TG/HDL-C < 2.97** **(*N* = 221)**	**TG/HDL-C ≥ 2.97** **(*N* = 311)**
**Composite endpoint of renal function decline**			
Number of cases, *n (%)*	35 (15.62)	46 (20.81)	69 (22.19)
Unadjusted, OR (95 % CI)	ref.	1.42 (0.87, 2.31)	1.54 (0.98, 2.41)
* P*		0.16	0.06
Age and gender adjusted, OR (95 % CI)	ref.	1.46 (0.89, 2.38)	1.69 (1.07, 2.68)
* P*		0.13	0.02
Multivariable-adjusted, OR (95 % CI)	ref.	1.32 (0.75, 2.30)	1.90 (1.21, 3.23)
* P*		0.33	0.02
**Decline in eGFR category**			
Number of cases, *n (%)*	33 (14.73)	42 (19.00)	62 (19.94)
Unadjusted, OR (95 % CI)	ref.	1.36 (0.82, 2.24)	1.44 (0.91, 2.29)
* P*		0.23	0.12
Age and gender adjusted, OR (95 % CI)	ref.	1.39 (0.84, 2.30)	1.57 (0.98, 2.51)
* P*		0.20	0.06
Multivariable-adjusted, OR (95 % CI)	ref.	1.33 (0.76, 2.32)	1.89 (1.12, 3.21)
* P*		0.32	0.02
**Rapid decline in eGFR**			
Number of cases, *n (%)*	24 (10.71)	32 (14.48)	47 (15.11)
Unadjusted, OR (95 % CI)	ref.	1.41 (0.80, 2.48)	1.48 (0.88, 2.51)
* P*		0.23	0.14
Age and gender adjusted, OR (95 % CI)	ref.	1.43 (0.81, 2.53)	1.57 (0.92, 2.67)
* P*		0.22	0.10
Multivariable-adjusted, OR (95 % CI)	ref.	1.24 (0.30, 5.13)	3.28 (0.97, 11.10)
* P*		0.16	0.06
**> 30 % decline in eGFR**			
Number of cases, *n (%)*	9 (4.02)	15 (6.79)	23 (7.40)
Unadjusted, OR (95 % CI)	ref.	1.74 (0.74, 4.06)	1.91 (0.87, 4.21)
* P*		0.20	0.11
Age and gender adjusted, OR (95 % CI)	ref.	1.74 (0.74, 4.06)	1.89 (0.86, 4.19)
* P*		0.20	0.12
Multivariable-adjusted, OR (95 % CI)	ref.	1.80 (0.70, 4.60)	2.56 (1.05, 6.38)
* P*		0.22	0.04

*OR* odds ratio; *CI* confidence interval; *eGFR* estimated glomerular filtration rate; *HDL-C* high-density lipoprotein cholesterol; *TG* triglycerides

*P* for trend in all multivariable-adjusted models are *P* < 0.05

^a^ Multivariable analysis was adjusted for age, gender, hypertension, diabetes or high blood sugar, obesity, waist circumference, smoking, drinking, history of heart disease and stroke, low-density lipoprotein cholesterol, eGFR category

^b^ Decline in eGFR category (≥ 90 [G1], 60–89 [G2], 45–59 [G3a], 30–44 [G3b], 15–29 [G4], < 15 [G5] mL/min/1.73 m^2^) was defined as certain decrease in eGFR category from baseline

^c^ Rapid decline in eGFR was defined as sustained decline in eGFR > 5 mL/min/1.73 m^2^ per year

^d^ 30 % decline in eGFR was defined as > 30 % decrease in eGFR from baseline

## Discussion

In the current study, all individuals were divided into the non-CKD and CKD group by a threshold of eGFR < 60 mL/min/1.73m^2^. Because in young adult men and women, the average eGFR value is approximately 125 mL/min/1.73m^2^, and a GFR < 60 mL/min/1.73m^2^ is less than half of that [24]. Previous studies showed that the risk of all-cause and cardiovascular mortality significantly increased when eGFR < 60 mL/min/1.73m^2^ in the normal population, a similar trend also founded in renal failure, CKD progression, and cardiovascular disease [25–27]. This study examination of the NRF and IRF groups indicated that the high TG/HDL-C ratio was an independent risk factor for the decline in renal function in both groups after adjusting for confounders.

### Comparisons with other studies and what does the current work add to the existing knowledge

Several previous prospective cohort studies reported associations between various measures of dyslipidemia with renal dysfunction. For example, Schaeffner et al. found that high TC/HDL-C, high non-HDL-C, TC, and low HDL-C were positively associated with increased risk of renal function decline in apparently healthy men [28]. Another study from American, which examined 12,728 participants with normal or mild impairment of renal function, also found that high TG and low HDL-C predicted a high risk of developing renal function decline [29]. Similar results were reported in community-based population studies in American and China and a national health screening study in Korea [30–32]. However, these studies focused on the relationship between the TG/HDL-C levels and increased risk of kidney dysfunction in participants with apparently normal kidney function and did not assess the individuals with impaired renal function at baseline. The present study compared individuals with NRF and IRF at baseline. Another longitudinal study from Japan found that higher TG/HDL-C levels were associated with the incidence and progression of CKD [33]. However, the reports of these associations in Chinese populations were limited. The current study results provided additional evidence that a higher TG/HDL-C ratio is an independent risk factor for the decline of renal function in the Chinese population.

### Potential mechanisms underlying the relationship of TG/HDL-C with renal function decline

Previous studies have proved that the relationship of TG/HDL-C with the decline in kidney function can be explained suggested by several potential mechanisms. First, the filtered proteins like albumin and lipoproteins contained phospholipids and cholesterol. These materials can stimulate tubulointerstitial, causing inflammation and injury in the reabsorption progress [34–36]. Second, the lipoproteins accumulating in the glomerular mesangium stimulate mesangial cells to generate matrix proteins and promote the production of proinflammatory cytokines, which recruit circulating macrophages and activate resident macrophages, culminating in glomerulosclerosis [34]. Third, a high TG/HDL-C ratio is significantly associated with elevated levels of small dense LDL-C particles in previous studies [37],which are highly atherogenic [38] and risk factors for CKD [39]. Fourth, TG/HDL-C is a reliable indicator of insulin resistance [12, 40], which induces oxidative stress [41]. Oxidative stress impairs the activation of nuclear factor erythroid-2-related factor-2, which protects against kidney tissue injury [42]. This study used a multivariable logistic regression model to adjust for diabetes, hypertension, obesity, stroke history, and heart disease history and found that the relationship of high TG/HDL-C ratio and the decline of eGFR remained significant, suggesting that the TG/HDL-C is an independent risk factor for eGFR decline.

### Study strengths and limitations

This study has several strengths. First, CHARLS adopted stratified, random, population-proportional-to-size sampling within the Chinese mainland. Thus, the population in this study was unbiased and representative, and the result of this study strongly supports the association of serum lipid parameters with eGFR decline in the general population in China. Second, the current study using different definitions of renal function decline to evaluate the association of TG/HDL-C and renal function, ensuring the generalizability of the results. Third, because this study was longitudinal, the development of lipid nephrotoxicity could be considered time-sequential to some extent.

There are also some limitations to this study. First, the 4-year follow-up time may have been insufficient to fully assess the impact of dyslipidemia on renal function. However, the large sample size probably reduced the impact of this limitation. Second, some potential risk factors were not adjusted, such as exercise history, use of hypolipidemic agents, and other relevant pharmacotherapies, because these data were only available for a small number of participants in the CHARLS study. Third, CHARLS mainly focuses on people over 45 years old, so the associations identified here should also be examined in a younger population before generalization. Despite these limitations, this study provides support for the importance of lowering the TG/HDL-C as a method to maintain kidney function in the elderly.

## Conclusions

This study demonstrated that a high TG/HDL-C ratio is an independent risk factor for reduced renal function in the Chinese population. These findings may help to provide clinical guidelines for the primary prevention of CKD. More convincing endpoint events of kidney disease, such as ESRD, should be adopted in future studies to further clarify the causal relationship between dyslipidemia and renal dysfunction. It is also essential to research the potential benefit of lipid lowering therapy in preventing or slowing the progression of renal disease.

## Supplementary Information

**Additional file 1:**

## Data Availability

The datasets used during the current study are available in the CHARLS, http://charls.pku.edu.cn/index/zh-cn.html.

## Abbreviations

BMI: Body mass index
CVD: Cardiovascular disease
CHARLS: China Health and Retirement Longitudinal Study
CKD: Chronic kidney disease
CKD-EPI: CKD Epidemiology Collaboration
eGFR: Estimated glomerular filtration rate
HbA1c: Hemoglobin A1c
HDL-C: High-density lipoprotein cholesterol
IRF: Impaired renal function
LDL-C: Low-density lipoprotein cholesterol
ESRD: End-stage renal disease
NRF: Normal renal function
SCr: Serum creatinine
TC: Total cholesterol
TG: Triglyceride
TG/HDL-C: Triglycerides to high-density lipoprotein cholesterol
