# Supplementary material for: The effect of triglycerides to high-density lipoprotein cholesterol ratio on the reduction of renal function: findings from China health and retirement longitudinal study (CHARLS)
Source: Lipids Health Dis. 2021 Sep 20;20:110. doi: 10.1186/s12944-021-01542-5 (PMC8454112; doi:10.1186/s12944-021-01542-5)
Supplement: Supplementary file 1 — Additional file 1: [file 12944_2021_1542_MOESM1_ESM.docx]

**Table S1. Baseline characteristics of study population by eGFR**

| **Variable** | **eGFR ≥ 60 mL/min/1.73 m^2^** | **eGFR <60 mL/min/1.73 m^2^** | **P** |
| --- | --- | --- | --- |
| Number, *n (%)* | 6,560 (89.67) | 756 (10.33) |  |
| Age, mean *(SD)*, years | 57.83 (8.61) | 66.48 (8.98) | <0.001 |
| Women, *n (%)* | 3,607 (54.98) | 394(52.12) | 0.13 |
| BMI, mean *(SD)*, kg/m^2^ | 23.66 (3.81) | 23.62 (4.31) | 0.41 |
| Waist circumference, mean *(SD)*, cm | 84.43 (12.36) | 85.74 (13.21) | 0.002 |
| Obesity, *n (%)* | 1,509 (23.00) | 174 (23.02) | 0.02 |
| Hypertension, *n (%)* | 2,439 (37.25) | 411 (54.58) | <0.001 |
| Diabetes, *n (%)* | 1,018 (15.52) | 158 (20.90) | <0.001 |
| Smoking, *n (%)* | 2,465 (37.66) | 323 (43.01) | 0.004 |
| Drinking, *n (%)* | 1,981 (30.26) | 232 (30.93) | 0.71 |
| History of stroke, *n (%)* | 125 (1.92) | 28 (3.74) | 0.001 |
| History of heart problems, *n (%)* | 769 (11.82) | 130 (17.33) | <0.001 |
| Hemoglobin A1c, mean (*SD*), (%) | 5.25 (0.78) | 5.33 (0.83) | <0.001 |
| LDL-C, mean *(SD)*, mg/dl | 116.09 (33.62) | 122.50 (36.40) | <0.001 |
| HDL-C, mean *(SD)*, mg/dl | 50.85 (14.43) | 50.02 (15.02) | 0.06 |
| TG, mean *(SD)*, mg/dl | 127.86 (79.21) | 140.29 (82.52) | <0.001 |
| TC, mean *(SD)*, mg/dl | 191.63 (37.03) | 200.59 (40.06) | <0.001 |
| TG/HDL-C, mean *(SD)* | 1.16 (0.29) | 2.21 (0.39) | <0.001 |
| Serum creatinine, mean *(SD)*, mg/dl | 0.74 (0.14) | 1.07 (0.21) | <0.001 |
| eGFR, mean *(SD)*, ml/min per 1.73m^2^ | 78.21 (8.59) | 52.02 (7.08) | <0.001 |

Continuous variables were expressed as the mean ± standard deviation (SD), and categorical variables were described as frequencies and percentages. Continuous variables were compared by Kruskal-Wallis test. Categorical variables were compared by ~~one-way ANOVA~~Chi-square test. eGFR, estimated glomerular filtration rate; LDL-C, low-density lipoprotein cholesterol; TG, triglycerides; TC, total cholesterol; HDL-C, high-density lipoprotein cholesterol; BMI, body mass index.

**Table S2. Basic characteristics of study population with eGFR ≥ 60 mL/min/1.73 m^2^ at baseline by TG/HDL-C ratio**

| **Variable** | **Group 1** | **Group 2** | **Group 3** | **P** |
| --- | --- | --- | --- | --- |
|  | **TG/HDL-C <1.60** | **TG/HDL-C 1.60-2.97** | **TG/HDL-C ≥2.97** |  |
| Number, *n (%)* | 2,229 (33.98) | 2,202 (33.57) | 2,129 (32.45) |  |
| Age, mean *(SD)*, years | 58.51 (9.03) | 57.84 (8.53) | 57.10 (8.18) | <0.001 |
| Women, *n (%)* | 1,154 (51.77) | 1,236 (56.13) | 1,217 (57.16) | <0.001 |
| BMI, mean *(SD)*, kg/m^2^ | 22.30 (3.41) | 23.66 (3.67) | 25.10 (3.83) | <0.001 |
| Waist circumference, mean *(SD)*, cm | 80.67 (10.50) | 84.58 (12.44) | 88.25 (12.90) | <0.001 |
| Obesity, *n (%)* | 349 (15.66) | 492 (22.34) | 668 (31.38) | <0.001 |
| Hypertension, *n (%)* | 653 (29.34) | 798 (36.27) | 988 (46.56) | <0.001 |
| Diabetes, *n (%)* | 206 (9.24) | 288 (13.08) | 524 (24.61) | <0.001 |
| Smoking, *n (%)* | 895 (40.22) | 789 (35.88) | 781 (36.82) | 0.01 |
| Drinking, *n (%)* | 775 (34.82) | 625 (28.42) | 581 (27.39) | <0.001 |
| History of stroke, *n (%)* | 32 (1.44) | 47 (2.14) | 46 (2.18) | 0.14 |
| History of heart problems, *n (%)* | 208 (9.40) | 240 (10.99) | 321 (15.23) | <0.001 |
| Hemoglobin A1c, mean (*SD*), (%) | 5.15 (0.60) | 5.22 (0.73) | 5.39 (0.96) | <0.001 |
| LDL-C, mean *(SD)*, mg/dl | 113.23 (29.81) | 120.70 (34.11) | 114.30 (36.29) | <0.001 |
| HDL-C, mean *(SD)*, mg/dl | 63.16 (13.13) | 49.99 (9.40) | 38.84 (8.15) | <0.001 |
| TG, mean *(SD)*, mg/dl | 68.49 (16.45) | 108.69 (23.66) | 209.86 (87.62) | <0.001 |
| TC, mean *(SD)*, mg/dl | 187.63 (33.88) | 190.48 (37.48) | 197.00 (39.04) | <0.001 |
| TG/HDL-C, mean *(SD)* | 1.11 (0.28) | 2.19 (0.39) | 5.72 (3.07) | <0.001 |
| Serum creatinine, mean *(SD)*, mg/dl | 0.73 (0.14) | 0.73 (0.14) | 0.75 (0.15) | <0.001 |
| eGFR, mean *(SD)*, ml/min per 1.73m^2^ | 78.60 (8.40) | 78.26 (8.60) | 77.76 (8.76) | 0.01 |

Continuous variables were expressed as the mean ± standard deviation (SD), and categorical variables were described as frequencies and percentages. Continuous variables were compared by Kruskal-Wallis test. Categorical variables were compared by ~~one-way ANOVA~~Chi-square test. eGFR, estimated glomerular filtration rate; LDL-C, low-density lipoprotein cholesterol; TG, triglycerides; TC, total cholesterol; HDL-C, high-density lipoprotein cholesterol; BMI, body mass index.

**Table S3. Basic characteristics of study population with eGFR < 60 mL/min/1.73 m^2^ at baseline by TG/HDL-C ratio**

| **Variable** | **Group 1** | **Group 2** | **Group 3** | **P** |
| --- | --- | --- | --- | --- |
|  | **TG/HDL-C <1.60** | **TG/HDL-C 1.60-2.97** | **TG/HDL-C ≥2.97** |  |
| Number, *n (%)* | 224 (29.63) | 221 (29.23) | 311 (41.14) |  |
| Age, mean *(SD)*, years | 67.33 (9.24) | 67.05 (8.52) | 65.46 (9.02) | 0.04 |
| Women, *n (%)* | 111 (49.55) | 111 (50.23) | 172 (55.31) | 0.34 |
| BMI, mean *(SD)*, kg/m^2^ | 22.42 (4.77) | 22.88 (3.95) | 25.04 (3.76) | <0.001 |
| Waist circumference, mean *(SD)*, cm | 82.04 (11.69) | 83.55 (14.25) | 90.04 (12.32) | <0.001 |
| Obesity, *n (%)* | 34 (15.18) | 51 (23.08) | 89 (28.62) | <0.001 |
| Hypertension, *n (%)* | 109 (49.10) | 121 (54.75) | 181 (58.39) | 0.11 |
| Diabetes, *n (%)* | 36 (16.07) | 39 (17.65) | 83 (26.69) | 0.004 |
| Smoking, *n (%)* | 104 (46.85) | 99 (44.80) | 120 (38.96) | 0.16 |
| Drinking, *n (%)* | 82 (36.94) | 62 (28.05) | 88 (28.66) | 0.07 |
| History of stroke, *n (%)* | 8 (3.60) | 11 (4.98) | 9 (2.94) | 0.47 |
| History of heart problems, *n (%)* | 31 (13.96) | 40 (18.18) | 59 (19.16) | 0.28 |
| Hemoglobin A1c, mean (*SD*), (%) | 5.27 (0.75) | 5.25 (0.69) | 5.43 (0.96) | 0.04 |
| LDL-C, mean *(SD)*, mg/dl | 117.50 (31.48) | 127.82 (34.36) | 122.33 (40.47) | 0.01 |
| HDL-C, mean *(SD)*, mg/dl | 64.43 (13.85) | 50.60 (9.93) | 39.23 (8.62) | <0.001 |
| TG, mean *(SD)*, mg/dl | 72.68 (18.63) | 110.46 (23.97) | 210.18 (84.21) | <0.001 |
| TC, mean *(SD)*, mg/dl | 194.76 (36.18) | 198.00 (39.36) | 206.63 (42.44) | 0.005 |
| TG/HDL-C, mean *(SD)* | 1.16 (0.29) | 2.21 (0.39) | 5.71 (3.10) | <0.001 |
| Serum creatinine, mean *(SD)*, mg/dl | 1.06 (0.20) | 1.07 (0.20) | 1.07 (0.22) | 0.71 |
| eGFR, mean *(SD)*, ml/min per 1.73m^2^ | 52.49 (6.75) | 51.95 (6.83) | 51.73 (7.48) | 0.54 |

Continuous variables were expressed as the mean ± standard deviation (SD), and categorical variables were described as frequencies and percentages. Continuous variables were compared by Kruskal-Wallis test. Categorical variables were compared by ~~one-way ANOVA~~Chi-square test. eGFR, estimated glomerular filtration rate; LDL-C, low-density lipoprotein cholesterol; TG, triglycerides; TC, total cholesterol; HDL-C, high-density lipoprotein cholesterol; BMI, body mass index.
